# Gallbladder Myeloid Sarcoma Associated With NPM1 Mutated Acute Myeloid Leukemia

**DOI:** 10.1002/jha2.70086

**Published:** 2025-06-30

**Authors:** Francesco Grimaldi, Maria Gabriella Rascato, Pier Paolo Mainenti, Marcella Giordano, Massimo Mascolo, Fabrizio Pane

**Affiliations:** ^1^ Department of Clinical Medicine and Surgery, Hematology Division University of Napoli “Federico II” Naples Italy; ^2^ CNR, Institute for Biostructure and Bioimages Naples Italy; ^3^ Department of Advanced Biomedical Sciences, Pathology Unit University of Napoli “Federico II” Naples Italy

**Keywords:** acute myeloid leukemia, extramedullary involvement, gallbladder, myeloid sarcoma

1

According to the 2022 World Health Organization (WHO) [1] and International Consensus Classification (ICC) [2] of myeloid neoplasm, myeloid sarcoma (MS) is a de novo disease characterized by extramedullary infiltration of immature myeloid precursors, that may accompany peripheral blood and marrow involvement of acute myeloid leukemia (AML), or present as relapse or as progression to AML of a prior myeloproliferative or myelodysplastic disorder. Diagnosis of MS is rare, and affects approximately 2%–10% of AML patients [3], with common sites including skin, lymph nodes, bones, and soft tissues [4]. Most of the cases present with a monoblastic or myelomonocytic infiltration [5], are associated with chromosomal abnormalities like t8;21, trisomy 8, or chromosome 7 partial or complete deletion [5], and can show recurrent mutations in *NPM1*, *FLT3*, and *N‐RAS* genes [6]. MS gastrointestinal involvement [4, 7] is less frequent (∼10%), predominantly affects the small or large intestine, and generally presents with symptoms like abdominal pain or bowel obstruction.

Herein, we describe an unusual case of gallbladder infiltration by MS in an elderly AML patient. An 81‐year‐old male was admitted to our clinic for AML with hyperleukocytosis (WBC 100,000/mm^3^, Hb 12.3 g/dL, and PLT 59,000/mm^3^). Bone marrow flow cytometry demonstrated 83% monocytic blasts (CD34−, CD117−, CD33+, CD13+, CD14+/−, CD64+, CD36+, CD4+, and CD56+). Standard cytogenetic with fluorescence in situ hybridization analysis revealed a normal karyotype, while next‐generation sequencing identified *ASXL1*, *SRSF2*, and *NPM1* type A mutations. A final diagnosis of AML with defining genetic abnormalities was done following the 2022 WHO criteria [1].

Given advanced age and low‐performance status, cytoreduction with hydroxyurea followed by decitabine [8] (5‐day schedule) was started. However, on day +20 patient developed fever (38.6°C), right upper quadrant abdominal pain, nausea, and elevation of alanine transaminase, gamma‐glutamyl‐transpeptidase, and alkaline phosphatase liver enzymes. Abdominal computed tomography showed a distended gallbladder with infundibular gallstones and possible choledocholithiasis. Supportive treatment, including hydration, fasting, and intravenous antibiotics (amikacin and tigecycline), was initiated. However, the patient's clinical condition rapidly deteriorated, prompting further evaluation.

Magnetic resonance cholangiopancreatography revealed a <1 cm common bile duct stone, mild biliary dilatation, and gallbladder wall thickening (Figure 1a). Laparoscopic cholecystectomy combined with endoscopic retrograde cholangiopancreatography for bile duct clearance was performed, resulting in fast clinical and biochemical improvement. Unexpectedly, histopathological examination of the gallbladder disclosed monocytic cell infiltrates throughout the wall (Figure 1b), immunoreactive for CD68 and aberrantly positive for cytoplasmic *NPM1a* (NPMc+), confirming leukemic infiltration (Figure 1c). Despite surgical management, blast recurrence occurred during hematologic recovery, and the patient succumbed to refractory disease after a second cycle of decitabine.

**FIGURE 1:**
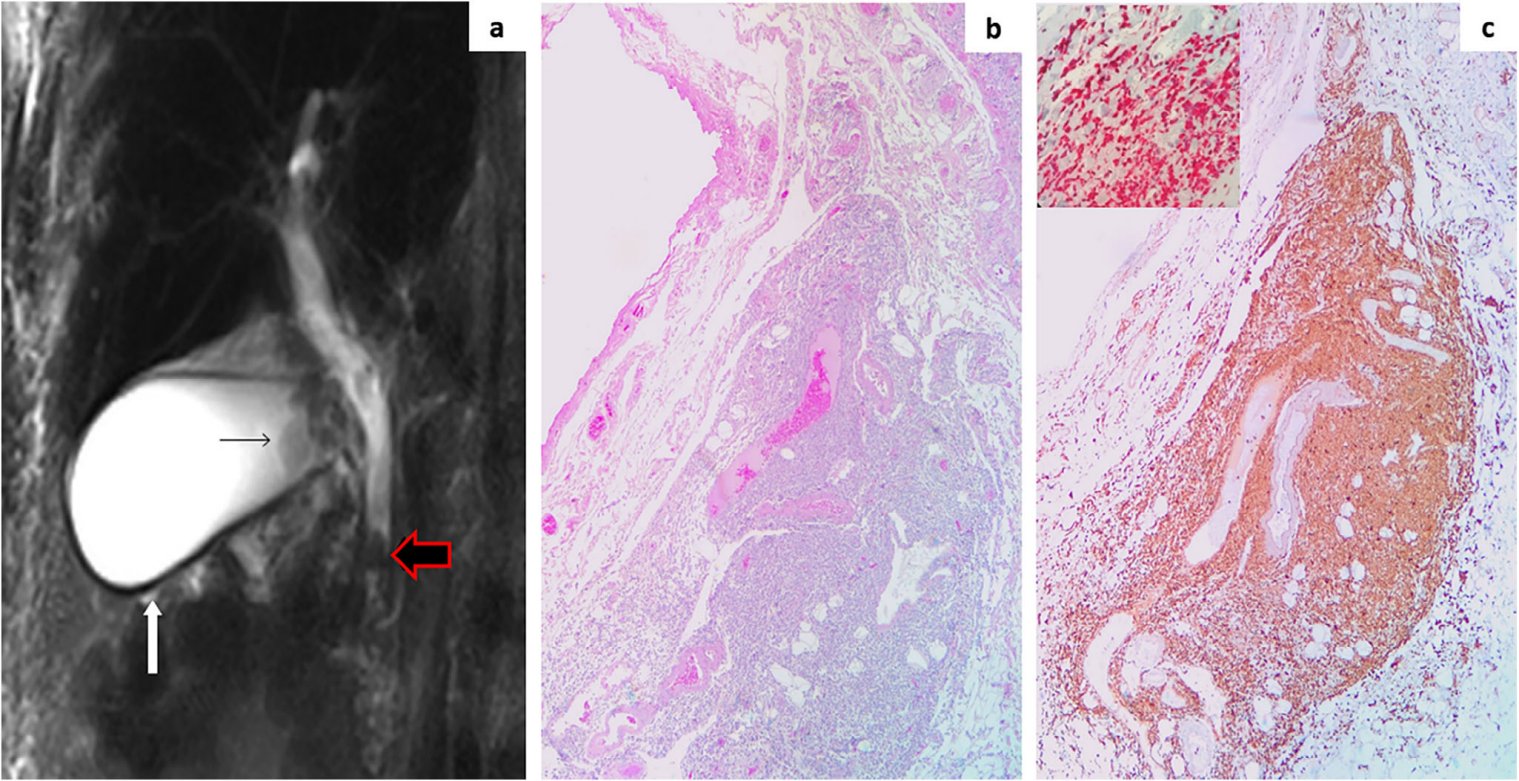
(a) Magnetic resonance cholangiopancreatography (MRCP) radial image shows a <1 cm stone in the common bile duct (red outline black arrow) with mild proximal biliary tree dilatation; the gallbladder shows distended lumen, mild wall thickening (white arrow) and gallstones in the infundibulum (thin black arrow). (b) Gallbladder histopathology shows a wall infiltrated mainly by medium‐sized neoplastic monocytic cells (hematoxylin and eosin, original magnification, x10). (c) The leukemic cells show positivity for CD68 (original magnification, x20) and exhibit aberrant cytoplasmic nucleophosmin (inset: anti‐NPM staining).

Gallbladder infiltration by MS is exceedingly rare, with only approximately 50 cases of hepatobiliary leukemic involvement reported in the literature [7]. Given the diagnostic challenges and poor prognosis associated with such localization, clinicians should maintain a high index of suspicion and consider this differential diagnosis when AML patients present with atypical abdominal symptoms. Timely intervention and early histological confirmation remain crucial and may guide management, although prognosis remains poor in refractory cases.

## Author Contributions

F G wrote the manuscript. F G and M G R provided care for the patient. P P M performed radiological study and interpretation. M G and M M performed histological and immunohistochemical analysis and results interpretation. F P supervised manuscript preparation and approved the final version of the manuscript.

## Conflicts of Interest

The authors declare no conflicts of interest.

## Ethics Statement

The authors have nothing to report.

## Consent

The authors have nothing to report.

## Clinical Trial Registration

The authors have confirmed clinical trial registration is not needed for this submission.

## Data Availability

Data are provided within the manuscript and are available from the corresponding author upon reasonable request.
